# Screening for resistance to four fungal diseases and associated genomic regions in a snap bean diversity panel

**DOI:** 10.3389/fpls.2024.1386877

**Published:** 2024-06-11

**Authors:** Ana Campa, Valérie Geffroy, Elena Bitocchi, Alicia Noly, Roberto Papa, Juan José Ferreira

**Affiliations:** ^1^ Plant Genetic Group, Regional Service for Agrofood Research and Development (SERIDA), Villaviciosa, Asturias, Spain; ^2^ Université Paris-Saclay, CNRS, INRAE, Université Evry, Institute of Plant Sciences Paris-Saclay (IPS2), Gif sur Yvette, France; ^3^ Université Paris-Cité, CNRS, INRAE, Institute of Plant Sciences Paris-Saclay (IPS2), Gif sur Yvette, France; ^4^ Department of Agricultural, Food and Environmental Sciences, Marche Polytechnic University, Via Brecce Bianche, Ancona, Italy

**Keywords:** *Phaseolus vulgaris*, resistance genes, anthracnose, powdery mildew, *Pythium ultimum*, white mold, genome-wide association mapping

## Abstract

Anthracnose, white mold, powdery mildew, and root rot caused by *Colletotrichum lindemuthianum*, *Scletorinia sclerotiorum*, *Erysiphe* spp., and *Pythium ultimum*, respectively, are among the most frequent diseases that cause significant production losses worldwide in common bean (*Phaseolus vulgaris* L.). Reactions against these four fungal diseases were investigated under controlled conditions using a diversity panel of 311 bean lines for snap consumption (Snap bean Panel). The genomic regions involved in these resistance responses were identified based on a genome-wide association study conducted with 16,242 SNP markers. The highest number of resistant lines was observed against the three *C. lindemuthianum* isolates evaluated: 156 lines were resistant to CL124 isolate, 146 lines resistant to CL18, and 109 lines were resistant to C531 isolate. Two well-known anthracnose resistance clusters were identified, the Co-2 on chromosome Pv11 for isolates CL124 and CL18, and the Co-3 on chromosome Pv04 for isolates CL124 and C531. In addition, other lesser-known regions of anthracnose resistance were identified on chromosomes Pv02, Pv06, Pv08, and Pv10. For the white mold isolate tested, 24 resistant lines were identified and the resistance was localized to three different positions on chromosome Pv08. For the powdery mildew local isolate, only 12 resistant lines were identified, and along with the two previous resistance genes on chromosomes Pv04 and Pv11, a new region on chromosome Pv06 was also identified. For root rot caused by *Pythium*, 31 resistant lines were identified and two main regions were located on chromosomes Pv04 and Pv05. Relevant information for snap bean breeding programs was provided in this work. A total of 20 lines showed resistant or intermediate responses against four or five isolates, which can be suitable for sustainable farm production and could be used as resistance donors. Potential genes and genomic regions to be considered for targeted improvement were provided, including new or less characterized regions that should be validated in future works. Powdery mildew disease was identified as a potential risk for snap bean production and should be considered a main goal in breeding programs.

## 1 Introduction

Snap bean (green bean or French bean) is a group of common beans (*Phaseolus vulgaris* L.) whose immature pods can be consumed as a vegetable. The origin of the snap bean is still uncertain. The very complex evolutionary history that characterizes *P. vulgaris*, including the spread and adaptation processes outside the centers of domestication (Bellucci et al., 2023), makes it difficult to obtain a clear picture. Snap beans may have arisen in Europe as a result of selective pressure on the pod characteristics of dry cultivars (Wade, 1937; Myers and Bagget, 1999). However, an independent derivation from dry beans may have also occurred in the Americas, with snap beans subsequently brought to Europe (Wallace et al., 2018). Most snap beans cultivated today are elite cultivars obtained through intensive breeding programs.

Plant diseases cause significant losses in food crop production worldwide (Ristaino et al., 2021; Singh et al., 2023). Anthracnose (ANT), white mold (WM), powdery mildew (PM), or root rot diseases are the most frequent fungal diseases present throughout bean production areas worldwide (Schwartz et al., 2005).

ANT is caused by the fungus *Colletotrichum lindemuthianum* (Sacc. & Magnus) Lams.- Scrib., a highly variable pathogen with several reported pathotypes (Padder et al., 2017). Resistance generally follows a qualitative mode of inheritance, with a very specific plant-pathogen interaction. Several ANT resistance genes (*Co-*) have been described and mapped on all the *P. vulgaris* chromosomes, except Pv05, Pv06, and Pv10 (Kelly and Vallejo, 2004; Ferreira et al., 2013; Campa et al., 2014);. Three chromosome regions including ANT resistance genes have been identified as complex clusters of resistant genes including closely linked race-specific genes, different allelic series of a *Co*- gene, or even genes conferring resistance to a diverse selection of pathogens (Kelly and Vallejo, 2004; Miklas et al., 2006; Ferreira et al., 2013; Meziadi et al., 2016). Some of these clusters have been physically positioned in the bean reference genome (Schmutz et al., 2014): the commonly known Co-1 cluster was located at the end position of chromosome Pv01, approximately at 50 Mbp (Richard et al., 2014; 2021; Vaz Bisneta and Gonçalves-Vidigal, 2020). On Pv04 a well-known anthracnose resistance cluster (David et al., 2008, David et al, 2009), usually identified as the Co-3 cluster, was located in a physical position of 1.28–2.04 Mbp (Murube et al., 2019; Vaz Bisneta et al., 2021). Finally, the first anthracnose resistance cluster identified by Creusot et al. (1999) was physically located at the end position of chromosome Pv11, around 45,15–48,66 Mbp (Chen et al., 2018). Using a genome-wide association study (GWAS) a broader range of genetic variation was explored, and new chromosome regions have been recently identified on chromosomes Pv02, Pv05, Pv06, and Pv10 (Wu et al., 2017; Banoo et al., 2020; Vidigal Filho et al., 2020; Almeida et al., 2021; Alvarez-Diaz et al., 2021; Costa et al., 2021; Vaz Bisneta et al., 2021; Simons et al., 2022).

The causal agent of WM in common bean is the fungus *Sclerotinia sclerotiorum* (Lib.) de Bary. Defense against this pathogen is conditioned by avoidance and physiological resistance mechanisms, both being quantitatively inherited (Miklas et al., 2001). Substantial progress has been made in understanding pathogenic variation, developing screening methods, understanding the genetics of resistance, and developing resistance-related molecular markers (Schwartz and Singh, 2013). Several quantitative trait loci (QTL) for resistance and avoidance have been identified based on bi-parental populations, with most having small to moderate effects, and located in all chromosomes except Pv10 (Schwartz and Singh, 2013; Vasconcellos et al., 2017; Escobar et al., 2022). Recently, three GWAS against WM were conducted and new chromosome regions associated with the resistance were identified, including chromosome Pv10 (Campa et al., 2020; Arkwazee et al., 2022; Escobar et al., 2022).

A broad range of genera and species of the order *Erysiphales* (Glawe, 2008) can cause PM. Two different species were identified as the common bean PM´s causal agent, *Erysiphe poligony* DC (Rezende et al., 1999; Schwartz et al., 2005), and *Erysiphe diffusa* (Cooke & Peck) U. Braun & S. Taka (Almeida et al., 2008). Genetic analyses have shown a qualitative mode of inheritance, with two independent epistatic genes (*Pm* genes) having been identified (Trabanco et al., 2012; Perez-Vega et al., 2013). One gene confers total resistance and masks the effect of the other gene which confers partial resistance. These genes have been located on chromosomes Pv04 (0,08–0,23 Mbp, candidate gene *Phvul.004G00150;* Campa and Ferreira, 2017) and Pv11, in a physical position corresponding to that of the ANT resistance cluster Co-2 (Perez-Vega et al., 2013). To date, one GWAS against PM has been conducted in common bean (Binagwa et al., 2021) and confirmed the locations of resistance genes on chromosomes Pv04, and Pv11, as well as identifying a new region on chromosome Pv10 (40,50–40,9 Mbp).

Root rot diseases can be caused by a complex group of fungi species, among which *Fusarium solani*, *Rhizoctonia solani* and *Pythium ultimum* are very frequent. *Pythium ultimum* (Py) is a soil pathogen that can cause seed rot before germination, pre-emergence and post-emergence damping-off, or root rot (Schwartz et al., 2005). Root rot resistance, including to Py, has been a main target trait for breeding programs in the East African region, where this disease is a primary cause of crop failures among subsistence farmers (Abawi and Pastor Corrales, 1990). Genetic resistance to this disease has been difficult to increase due to (i) its negative association with agronomically accepted white-seeded cultivars; (ii) the polygenic nature of this resistance, being identified as having qualitative and quantitative inheritance; (iii) and its linkage with the *P* gene responsible for the white seed color (McClean et al., 2018) or with undesirable traits, such as late maturity, low yield potential, and poor pod quality (Deakin, 1974; Schwartz et al., 2005; Campa et al., 2010). White-seeded cultivars with high levels of resistance have not been identified, but within colored seeds, two QTL associated with the emergence rate and with seedling vigor were identified in linkage groups Pv03 and Pv06 (Campa et al., 2010). A GWAS against Py (Dramadri et al., 2020) led to the identification of new genomic regions associated with this resistance on chromosomes Pv01 (6,27 Mbp), Pv02 (25,48 and 44,90 Mbp), Pv04 (41,96 Mbp), Pv05 (29,48 Mbp), and Pv09 (6,25 Mbp).

The control of plant diseases using classical pesticides raises serious concerns about food safety, environmental quality, and pesticide resistance. In the context of sustainable agriculture, which aims to reduce the environmental and climatic impact of primary production, or in the context of the organic farming system, which aims to use only inputs of natural origin, the control of diseases through plant breeding is the most effective strategy. The objective of this work was to investigate the reactions against four fungal diseases, ANT, WM, PM, and root rot caused by Py in a set of 311 snap bean lines (Snap Bean Panel-SBP; García-Fernández et al., 2022) to identify the most resistant materials that will be the more resilient under sustainable farming conditions. A GWAS was also conducted against each of these diseases to identify the genomic regions involved in the resistance. The results provide relevant information regarding the need for snap bean breeding programs in the snap bean market.

## 2 Material and methods

### 2.1 Plant material

The SBP contains 311 lines derived from accessions for snap consumption collected in European gene banks, working collections, and seed companies (Ferreira et al., 2021a, Ferreira et al., 2021b; García-Fernández et al., 2022; **Supplementary Table S1**). A set of 14 common bean lines derived from elite bean varieties including well-known resistance sources against the different diseases, was also used (**Supplementary Table S2**).

The 311 SBP lines were morphologically characterized in the field by García-Fernández et al. (2022) recording two qualitative plant traits (growth habit, wing flower color, standard flower color), four qualitative pod traits (pod length, pod cross-section, pod color, curvature) and six qualitative seed traits (primary seed color, secondary seed color, shine, longitudinal shape, transversal shape, seed size).

### 2.2 Resistance test

#### 2.2.1 ANT

Monosporic isolates CL18 and CL124 from local bean crops in northern Spain are maintained at the SERIDA pathogen collection (Ferreira et al., 2008). Monosporic isolate C531 is maintained at the Orsay fungal library and was collected in Costa Rica (Geffroy et al., 1999). Resistance tests were conducted according to standardized resistance tests (Schwartz et al., 1981 Each isolate was evaluated independently in separate experiments, CL18 and CL124 were evaluated at the SERIDA and C531 at the INRAE. Each experiment consisted of all SBP and check lines, with 8–10 seedlings per line sown in trays randomly distributed in the greenhouse. Plant responses were recorded per line 7 days after inoculation. Plants without visible symptoms or showing limited necrotic lesions were scored as resistant, R. Plants with large, sporulating lesions and dead plants were scored as susceptible, S (Van Schoonhoven and Pastor-Corrales, 1987). Resistant lines and those showing unclear reactions were evaluated in a second resistance test.

#### 2.2.2 PM

A local isolate of PM is preserved at the SERIDA, on plants of the S bean susceptible cultivar Xana, grown in spore-proof chambers. Resistance tests were conducted following Trabanco et al. (2012). Two independent resistance tests were carried out in the greenhouse, each one including half of the SBP and the check lines. In each resistant test, two pots per line were sown, with 4–5 plants per pot, randomly distributed in the greenhouse. Lines showing resistant and unclear responses were confirmed in a third resistance test. Plant responses were recorded per line 8–10 days after inoculation as infection type (IT) following a 0–4 scale: plants showing the IT0 response were considered R, plants showing IT1, IT2, or IT3 were considered intermediate (I), and plants showing IT4 were considered S (Trabanco et al., 2012).

#### 2.2.3 WM

The local isolate, WM2, maintained at the SERIDA pathogen collection was used (Pascual et al., 2010). Resistance tests were conducted using the straw method (Petzoldt and Dickson, 1996) and the same experimental design as that used with PM. Disease progression was evaluated 8–10 days after inoculation based on the level of invasion of the main stem, using a 1–9 severity scale, where 1 represents no symptoms and 9 represents an invasion of the third node and plant death. Because WM resistance is quantitatively inherited, each plant in the pot was rated separately and the disease value per pot was calculated as the arithmetic mean of the 4–5 plants. The disease value per line was calculated as the arithmetic mean of the two pots. Outliers were identified using the coefficient of variation (coefficient of variation = (standard deviation/mean) *100). Values equal to or less than 4.5 were considered highly R, values between 4.5 and 7 were considered I, and values above 7 were considered S.

#### 2.2.4 Py

A local Py isolate is maintained at the SERIDA pathogen collection on infected soil maintained at 4°C. Resistance tests were conducted in accordance with Campa et al. (2010). Two independent resistance tests were carried out, each one including half of the SBP and the check lines randomly distributed in the greenhouse. For each line, 10 seeds were inoculated with the fungus and 10 seeds (used as a control) were inoculated with agar plugs only. Disease assessment was evaluated 15 to 18 days after planting (first trifoliate leaf opened) as follows:


$$\text{\% germination =}\frac{\text{number of emerged inoculated seedlings x }100\;}{\text{number of emerged control seedlings}}$$

Values of 100% of germination were considered highly R, between 90%-30% of germination were considered I, and values equal to or less than 30% of germination were considered S.

### 2.3 Genotyping

A total of 311 SBP lines were genotyped using the Genotyping by Sequencing (GBS) method (Elshire et al., 2011) optimized following Schröder et al. (2016), based on the use of two enzymes (Taqα1 and MseI). Genomic DNAs from the lines were digested individually with the restriction enzyme combination and post-PCR purified with AMPure XP Beads. Paired-end library sequencing was carried out using the Illumina platform. For the bioinformatics analyses carried out for mapping and variant calling. Cutadapt v3.2 (Martin, 2011) was used to filter and trim low-quality reads and FastQC (Andrews, 2010) was used to check the final quality of the reads. Data from line SBP365 were not available. Each cleaned library was then mapped to the G19833 *P. vulgaris* v2.0 reference genome (Schmutz et al., 2014) using BWA-mem v0.7.15 (Li, 2013). The Genome Analysis ToolKit v4.1.9.0 (McKenna et al., 2010) was used for variant calling. Duplicated reads were sorted and filtered with Picard (2022) v2.4.1 (http://broadinstitute.github.io/picard) for sequencing duplicates. Variants were then discovered using “HaplotypeCaller” mode for each accession separately. Then the joint genotyping across all accessions was performed using “GenotypeGVCFs” mode. SNPs were extracted using the “SelectVariants” mode and pre-filtered using the “VariantFiltration” mode with the recommended parameters for hard filtering (–filterExpression “ QUAL< 60 || QD< 2.0 || MQ< 40.0 || FS > 60.0 || SOR > 3.0 || MQRankSum< -20.0 || ReadPosRankSum< -8.0”). SNPs were named based on their physical positions in the bean chromosomes (Pv) and their genomic positions in bp.

### 2.4 GWAS

SNPs were filtered using Tassel v5.2 software (Bradbury et al., 2007) for missing values (< 20%) and minor allele frequency (MAF> 0.05). A total of 16,242 SNPs were considered. The distribution of the SNPs along the 11 chromosomes was visualized with the “CMplot” package of the R project (R core Team 2022). GWAS was conducted based on the multi-locus model FASTmrEMMA (Wen et al., 2018) using the mrMLM package of the R project (Zhang et al., 2020; R Core Team, 2022). A Principal Component Analysis for two components and a Kinship matrix, obtained by the centered-IBS method, were considered to account for multiple levels of relatedness within the lines included in the panel. FASTmrEMMA includes a multi-locus model for true SNP detection at LOD≥ 3 from the associations showing p-values ≤ 0.005 (-log10(p) ≥ 2.3).

## 3 Results

### 3.1 Responses to four pathogens

**Figure 1:**
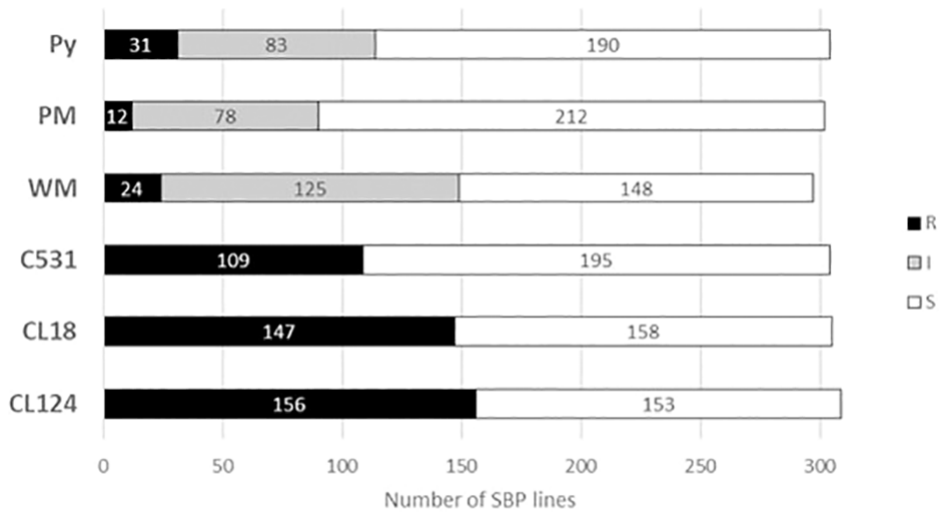
Stacked bar graph comparing the number of lines identified as R (resistant), I (intermediate) or S (susceptible) for each disease using the SBP. CL124, CL18, and C531: *Colletotrichum lindemuthianum* isolates. WM, white mold. PM, powdery mildew. Py, *Pythium ultimum* isolate.

The responses of the 311 SBP lines against the 6 isolates are summarized in **Figure 1** (see **Supplementary Table S1**). The highest number of R lines was observed against the three ANT isolates: 156, 146, and 109 lines were R to CL124, CL18, and C531, respectively. For WM, 24 lines were considered R, 125 lines were I, and 148 were S. For PM, only 12 lines were R, 78 were I, and most lines, 212, were S. For Py, 31 lines were considered R (all with colored seed), 83 were I, and 190 were S.

The evaluation of the six isolates was jointly considered in 282 lines (**Supplementary Table S1**). Considering the response to the three ANT isolates, 150 lines showed resistance to at least two ANT isolates, 53.3% of which were R to the three isolates, 35.3% to CL124 and CL18, 6.0% to CL124 and C531 and 5.3% to CL18 and C531. For resistance to ANT isolates and PM, 78% of the PM-R lines were also R to C531, and 89% were also R to CL18 and CL124. Considering responses to the ANT isolates and WM, 16%, 21%, and 33% of the lines showing R to WM were also R to CL18, C531, and CL124 isolates, respectively. Considering responses to Py and the ANT isolates, 17% of the lines were R to Py and CL124, and 10% were R to Py and CL18 and C531 isolates. No lines were R to WM and to PM, nor to PM and Py, and only 12% of the lines showed resistance against WM and Py.

### 3.2 Morphological characterizations of the most promising lines

In total, 20 lines were considered the most promising materials of the SBP based on their R or I responses against more than three isolates (**Table 1**). Following the results of García-Fernández et al. (2022), the morpho-agronomic traits of these lines are shown in **Table 1** (**Supplementary Table S1**). Most lines showed determinate growth habits, and only five showed indeterminate climbing habits. For pod color, the most frequent phenotype was green or green-mottled color, with only one line having yellow pods (SBP372) and one line having purple pods (SBP096). Most lines (11) showed colored seeds, with only single lines having either red or black seeds, and nine lines were white-seeded. Different pod and seed sizes were homogeneously distributed (**Table 1**).

**Table 1:** Morpho-agronomic characterization of the SBP lines showing resistance or intermediate response to more than three isolates.

Line	Diseases						Morpho-agronomic traits				
	Anthracnose						Growth	Pod	Pod	Primary Seed	Seed
	CL124	CL18	C531	WM	PM	Py	habit	color	length/section	color	size
SBP026	R	R	R	S	I	I	determinate	green	short/flat	cream	medium
SBP036	R	R	R	R	S	I	indeterminate	green	long/flat	dark brown	large
SBP043	R	R	R	S	I	I	determinate	green	medium/eight	brown	small
SBP082	S	S	R	R	S	R	indeterminate	green	medium/flat	red	medium
SBP096	R	R	R	I	S	I	indeterminate	purple	very long/flat	cream	large
SBP126	R	R	S	I	I	R	indeterminate	green mottled	long/flat	brown	medium
SBP145	R	R	R	S	R	S	determinate	green	medium/eight	white	very small
SBP166	R	R	S	I	I	I	determinate	green mottled	very long/pear	cream	medium
SBP186	R	R	S	I	I	I	determinate	green mottled	very long/pear	cream	medium
SBP190	R	R	R	S	R	S	determinate	green	short/round	white	small
SBP193	R	R	R	S	R	S	determinate	green	short/pear	white	medium
SBP195	R	R	R	I	I	S	determinate	green	short/pear	white	small
SBP200	R	R	R	S	I	I	determinate	green	short/eight	cream	small
SBP209	R	R	R	S	R	S	determinate	green	short/pear	white	small
SBP210	R	R	R	S	R	S	determinate	green	short/pear	white	small
SBP211	R	R	R	S	R	S	determinate	green	short/eight	white	small
SBP240	R	R	R	I	R	S	determinate	green	short/pear	white	small
SBP267	R	R	R	I	I	S	indeterminate	green	very long/flat	white	medium
SBP318	R	R	R	I	S	I	determinate	green	short/flat	brown	small
SBP372	R	R	S	I	I	R	determinate	yellow	medium/flat	black	small

R, resistant; I, intermediate; S, susceptible. CL124, CL18, C531, *Colletotrichum lindemuthianum* isolates WM, white mold; PM, powdery mildew; Py, *Pythium ultimum*.

### 3.3 GWAS

**Figure 2:**
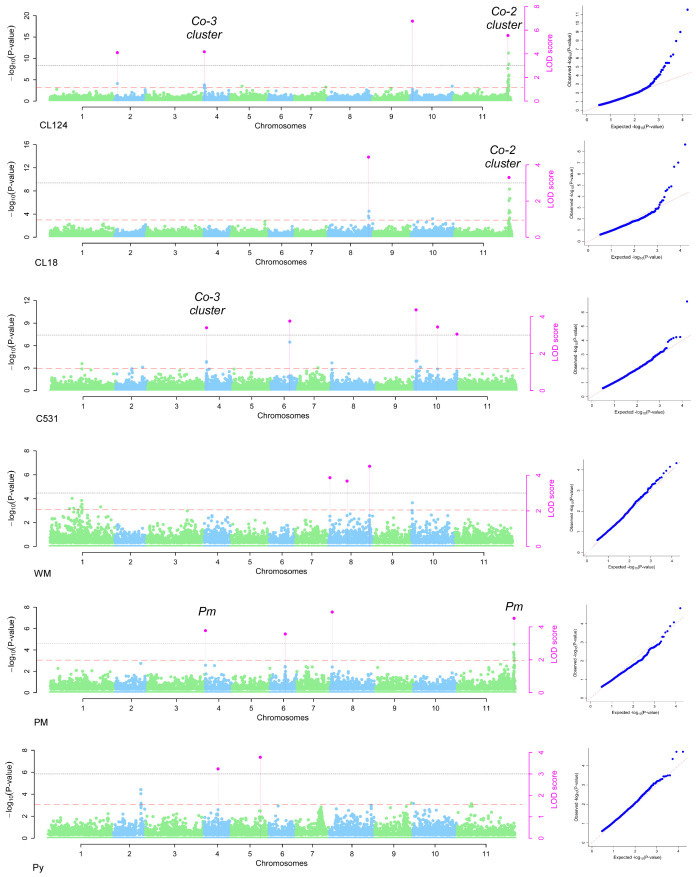
Manhattan and QQ plots obtained in the GWAS conducted in the SBP for anthracnose isolates (CL124, CL18, C531), white mold (WM), powdery mildew (PM), and *Pythium ultimum* (Py). The two threshold lines considered in the FASTmrEMMA method are indicated, in orange color for -log(p)≥ 2.3 and in grey color for LOD score ≥ 3. Pink dots indicate the SNPs significantly associated with each isolate. Correspondence with the previously described Co-2 and Co-3 clusters and with the *Pm* genes is indicated.

**Figure 3:**
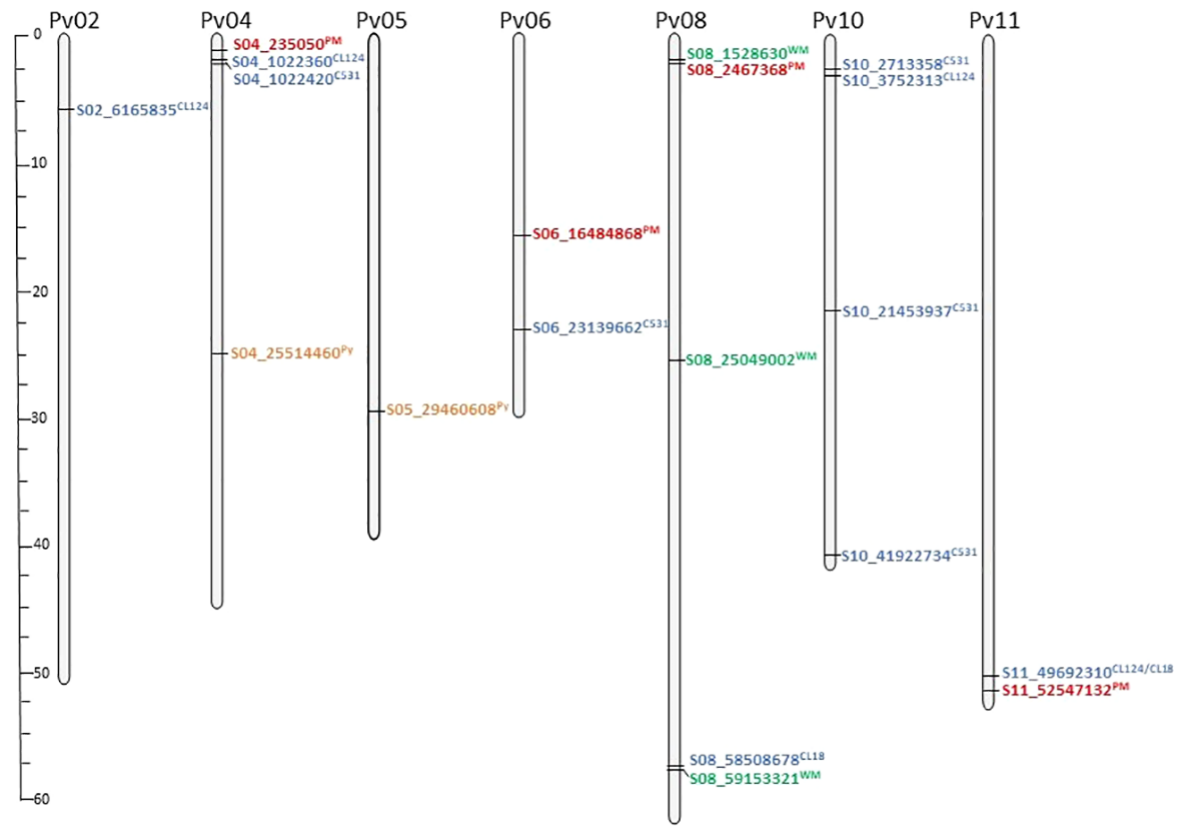
SNPs per chromosome associated with the different isolates evaluated in the Snap Bean Panel: anthracnose isolates (CL18, CL124, C531; blue color) white mold (WM; green color), powdery mildew (PM; red color), and *Pythium ultimum*. (Py; orange color). The specific isolate associated with each SNP is indicated in superscript. Scale bar represents chromosome size in Mbp.

The 16,242 SNPs considered after filtering were homogeneously distributed across the 11 bean chromosomes (**Supplementary Figure S1**). The GWAS conducted in the SBP led to the identification of 20 significantly associated SNPs on chromosomes Pv02, Pv04, Pv05, Pv06, Pv08, Pv10, and Pv11 (**Figures 2**, **3**; **Table 2**). Four SNPs were identified for the ANT isolate CL124 on chromosomes Pv02 (S02_6165835), Pv04 (S04_1022360), Pv10 (S10_3752313), and Pv11 (S11_49692310). For the ANT isolate CL18, two SNPs were identified, one at chromosome Pv08 (S08_58508678) and the other at Pv11 (S11_49692310), at the same position as for CL124. For the ANT isolate C531, five SNPs were identified on chromosomes Pv04 (S04_1022420), Pv06 (S06_23139662), and Pv10 (S10_2713358, S10_21453937, and S10_41922734). For the WM isolate, three SNPs were identified on chromosome Pv08 (S08_1528630, S08_25049002, S08_59153321). For PM, four SNPs were identified on chromosomes Pv04 (S04_235050), Pv06 (S06_16484868), Pv08 (S08_2467368), and Pv11 (S11_52547132). The SNP S08_2467368 was considered a false positive because it was associated with the allele N, which corresponded to a missing value (**Table 2**). Finally, two SNPs were identified for Py on chromosomes Pv04 (S04_25514460) and Pv05 (S05_29460608).

**Table 2:** SNPs that were significantly associated with each isolate in the GWAS performed with the FASTmrEMMA method and using the SBP.

Isolate	SNP	Chr	SNP effect	-log10(P)	LOD	r2 (%)	MAF	Genotype code 1
CL124	S02_6165835	Pv02	1.62	4.85	4.10	2.74	0.31	C
CL124	S04_1022360	Pv04	1.65	4.93	4.17	2.71	0.28	C
CL124	S10_3752313	Pv10	2.72	7.62	6.76	5.27	0.19	G
CL124	S11_49692310	Pv11	2.40	6.37	5.55	7.90	0.46	A
CL18	S08_58508678	Pv08	1.57	5.20	4.43	3.28	0.48	C
CL18	S11_49692310	Pv11	1.62	4.02	3.30	3.59	0.45	A
C531	S04_1022420	Pv04	1.45	4.12	3.40	2.47	0.28	C
C531	S06_23139662	Pv06	2.56	4.50	3.76	3.08	0.12	A
C531	S10_2713358	Pv10	2.68	5.14	4.37	5.55	0.18	G
C531	S10_21453937	Pv10	-2.22	4.16	3.44	3.13	0.15	G
C531	S10_41922734	Pv10	1.85	3.75	3.06	3.72	0.26	C
WM	S08_1528630	Pv08	0.00	4.61	3.86	0.00	0.18	T
WM	S08_25049002	Pv08	0.41	4.41	3.68	5.49	0.24	G
WM	S08_59153321	Pv08	0.33	5.28	4.51	5.11	0.42	T
PM	S04_235050	Pv04	0.41	4.51	3.77	4.04	0.12	C
PM	S06_16484868	Pv06	0.30	4.30	3.57	5.14	0.34	T
PM	S08_2467368	Pv08	0.38	5.69	4.90	8.66	0.37	N
PM	S11_52547132	Pv11	0.50	5.30	4.52	6.93	0.15	G
Py	S04_25514460	Pv04	-0.34	3.95	3.24	4.60	0.31	A
Py	S05_29460608	Pv05	-0.47	4.52	3.78	3.76	0.13	T

CL124, CL18, C531: anthracnose isolates; WM, white mold; PM, powdery mildew; Py, *Pythium ultimum*.

## 4 Discussion

The SBP includes a wide diversity of genotypes collected from European gene banks, working collections, and seed companies, and it can be considered to be representative of the level of diversity in European snap beans (García-Fernández et al., 2022). Disease resistance is a relevant goal for the adaptation of snap bean crops to more sustainable agriculture, which aims to reduce the environmental and climatic impact of primary production. The control of diseases through plant breeding is the most effective strategy, but further knowledge is still required to meet this objective. Improving our understanding of resistance mechanisms and the identification of new R genes are important to mitigate the risk of future disease outbreaks related to climate scenarios that threaten food security in many areas of the world (Ristaino et al., 2021; Singh et al., 2023).

The largest number of R lines was observed against the three ANT isolates evaluated, CL18, CL124, and C531. Traditionally, major achievements in snap bean breeding programs have been the modification of pod traits, seed sizes, and yield traits, but also resistance to diseases. ANT is regarded as a serious disease of common bean due to its seed-borne nature and pathogenic variability (Schwartz et al., 2005). Breeding to protect common bean against ANT has been intense since the beginning of the 20th century, therefore, it is not surprising to identify a high prevalence of resistance in the SBP.

In the GWAS for ANT isolates CL124 and CL18, the same SNP at the end of chromosome Pv11 was identified (S11_49692310). In common bean, this chromosomal region has been described as a complex cluster of R genes encoding nucleotide-binding leucine-rich repeat proteins (NB-LRR) that protect against different diseases or different strains of the same pathogen (Creusot et al., 1999; Campa et al., 2014; Meziadi et al., 2016). One of the first ANT resistance genes identified in common bean, the *Co-2* gene, has been mapped to this region (Mastenbroek, 1960). This was the first ANT resistance locus to be ‘tagged’ with molecular markers (Adam-Blondon et al., 1994), and has been extensively used in breeding programs since 1960 (Mastenbroek, 1960; Ferreira et al., 2012). For this reason, it was highly represented within the SBP lines, with several of them being derived from commercial varieties. Isolates CL124 and CL18 correspond to race 3 and race 38, respectively (Ferreira et al., 2008). ANT resistance genes against these two races have been located previously in the Co-2 cluster using different R genotypes (Rodriguez-Suarez et al., 2007; Campa et al., 2011, Campa et al, 2014). This is in agreement with the results obtained in the present work. For ANT isolate C531, the SNP located at Pv04 is close (60 bp apart) to the SNP identified for CL124 isolate, therefore they may correspond to closely linked genes of the same cluster. In this region of the Pv04 chromosome, the ANT resistance cluster Co-3 has also been located (Meziadi et al., 2016; Murube et al., 2019). The *Co-3* gene, originally known as *Mexique1*, was first described by Bannerot (1965) and was one of the first loci having described alleles that conditioned resistance to ANT. The breeding value of *Co-3* has been described as very specific for certain locations and was proposed to be included in pyramiding breeding programs (Kelly and Vallejo, 2004). One response to the short-term protection conferred by single genes is to pyramid specific R genes into one plant (Mundt, 1990). Here, the genes *Co-2* and *Co-3* were identified against the isolate CL124 and have probably been introgressed and pyramided in different breeding programs against ANT. Especially remarkable is the implication of chromosome Pv10 in the resistance to isolate C531 at physical positions 2.71, 21.5, and 41.92 Mbp. The Pv10 chromosome has not been previously identified in the resistance to ANT when genetic analyses were conducted using biparental populations. However, since 2016, the use of GWAS in the study of ANT resistance has led to the exploration of a broader range of genetic variation and the identification of new chromosomal regions (Zuiderveen et al., 2016). Recently, using GWAS, genomic regions on Pv10 were associated with ANT resistance (Banoo et al., 2020; Vidigal Filho et al., 2020; Simons et al., 2022), which is in agreement with the results obtained here. Furthermore, Wu et al. (2017) identified NB-LRR genes on chromosome Pv10 that are related to resistance against different diseases, including ANT. Other lesser-known regions for ANT resistance were identified in this work on chromosomes Pv02 (6.16 Mbp), Pv06 (23.13 Mbp), and Pv08 (58.50 Mbp) for CL124, C531, and CL18 isolates, respectively. At the start position of chromosome Pv02 (4.77 Mbp), an association with ANT resistance was recently identified and could correspond with the association identified here (Banoo et al., 2020). The region identified on chromosome Pv08 for the CL18 isolate may correspond to the one identified by Almeida et al. (2021) at 58.76 Mbp. In summary, some of the genomic regions identified in this work as being associated with resistance against ANT were previously identified in other GWAS. They represent minor R regions that could have important breeding values and should be validated in future works.

WM is endemic and widespread in North and South American countries, many of which have developed intensive breeding programs against the disease. The development of R bean cultivars against WM has been slow owing to the low number of R sources identified and the complex nature of the resistance, having both physiological and avoidance mechanisms. In this work, 24 lines were R to WM, and they can be used as R sources in snap breeding programs. Among them, only five lines were derived from commercial varieties (**Supplementary Table S1**), highlighting the importance of characterizing different genetic resources, such as landraces and/or wild materials, to exploit their diversity in breeding.

Three regions involved in the resistance to WM were identified on chromosome Pv08 (1.52, 25.04, 59.15 Mbp). Previously, several QTL and one meta-QTL (WM8.3; 37.32–46.73 Mbp) were identified on chromosome Pv08 based on biparental populations and GWAS (Vasconcellos et al., 2017; Campa et al., 2020). However, the physical positions of the regions identified in the present work do not correspond to those identified previously. The results may be difficult to compare owing to the different methods (fields or controlled conditions) used for the WM evaluations. In general, it has been assumed that WM evaluations under controlled conditions reveal physiological resistance, whereas field evaluations measure physiological resistance and disease avoidance mechanisms (Miklas et al., 2001). These new R regions could have important breeding values and should be validated in future works.

A low frequency of resistance against PM was observed, with only 12 SBP R lines identified. PM is a widespread plant disease with a rapidly increasing geographical distribution (Glawe, 2008). This expansion has been related to global climate change, because PM pathogen populations evolve quickly, showing high rates of variation due to the coexistence of sexual and asexual stages and the high dispersal capability. Here, the low frequency of observed resistance indicates the risk that can arise from this disease in the snap bean market and in sustainable farming systems. Increasing the resistance to PM should be a major goal of future breeding programs. The GWAS revealed three regions for PM resistance on chromosomes Pv04 (0.23 Mbp), Pv06 (16.49 Mbp), and Pv11 (52.54 Mbp). The regions on Pv04 and Pv11 corresponded with previously identified and mapped major resistance genes for PM (*Pm* genes; Trabanco et al., 2012; Perez-Vega et al., 2013; Campa et al., 2014). These regions at the beginning of chromosome Pv04 and the end of chromosome Pv11 are close to the physical positions of ANT clusters Co-3 and Co-2, respectively. This could explain the strong association identified in this work between the responses to PM and ANT isolates. However, the low number of R lines identified against PM, in contrast to the high number of ANT-R lines identified, also suggests that the genes conferring resistance against the pathogens are different. In fact, these two regions correspond to large clusters of NB-LRR genes, the major class of disease-resistance genes in plants (Meziadi et al., 2016). The new region identified on chromosome Pv06 (16.49 Mbp) against PM should be validated because it could have an important breeding value.

For Py disease, only 31 SBP lines showed high levels of resistance, all of them having colored seeds. Among the R lines, five were derived from commercial lines, whereas the remaining were derived from landraces or from unknown origins. Thus, they could be new interesting R sources to be exploited in snap bean breeding programs. The GWAS identified two regions on chromosomes Pv04 (25.51 Mbp) and Pv05 (29.46 Mbp). The physical position of the region identified on Pv05 corresponds to the one identified by Dramadri et al. (2020), confirming that this region plays a role in the genetic control of Py resistance. The other region at Pv04 was identified for the first time and should be confirmed in future works. Two other chromosomal regions on Pv02 and Pv07 have not been identified as significantly associated by the FASTmrEMMA method but are noteworthy regions showing log10(p) ≥ 3. On Pv02, a region between 43.92 and 44.21 Mbp was tagged with four SNPs having high -log(p) values in the GWAS: SNP S02_43921938 - log10(p)=4; S02_44213587 - log10(p)=4; S02_44217242 - log10(p)=3; S02_44217294 - log10(p)=4. This region was also identified as being associated with Py resistance in the GWAS conducted by Dramadri et al. (2020). Py resistance is often associated with colored seeds, and at this position of chromosome Pv02, between 33.7–48.5 Mbp, was located the seed coat color gene *B*, responsible for gray-brown colors and proposed to regulate simultaneously the flavonoid (color) and isoflavonoid (resistance) pathways (Beninger et al., 2000; Hagerty et al., 2016; Campa et al., 2023). Another seed coat color gene, the *Rk*, was located at this position, linked to *B*. *Rk* was described to be involved in the synthesis of red or garnet brown seed colors, and was proposed as a modifying color gene that affects the amount and type of tannins produced in the seed coat (Prakken, 1972). The results obtained in this work suggest that the seed coat color genes *B* or *Rk* could be involved in the resistance against Py. On Pv07, two SNPs showed high log(p) values (S07_28657174, log(p)=3; S07_28657186, log(p)=3) in a position corresponding to that of *P* gene (candidate gene *Phvul.007G171333*; location 28,752,131.28,766,155 bp; McClean et al., 2018) responsible for white seed color and linked to Py response (Deakin, 1974; Campa et al., 2010). White-seeded lines showing high levels of resistance have not been identified. Lines combining total or I resistance to WM and PM, or to PM and Py, have not been identified among all the isolates evaluated. This indicates that such resistance through breeding requires the pyramiding of genetic resistance to different pathogens and the introgression of resistance in specific pod phenotypes or snap bean market classes. For instance, in the groups of lines having flat cross-sections and very long green-colored pods (Type Romano), round cross-sections, or long yellow-colored pods (Yellow wax), a lack of genotypes R to PM was detected (**Supplementary Table S1**). In total, 20 lines were identified as the most promising, showing R or I responses to more than three isolates. Among them, the line SBP240 is very promising, with resistance to ANT and PM and moderate resistance to WM. This set of 20 lines provides different genotypes with high levels of resistance and wide phenotypic diversity that can be used in sustainable farm production systems with low input needs. These lines are also potential resistance sources for snap bean breeding programs. The data revealed by the GWAS can help breeders to identify useful gene/s or genomic regions for targeted breeding programs.

## 5 Conclusions

This work led to the identification of the most promising snap bean lines for sustainable farm production and potential resistance sources for breeding programs focused on developing varieties with broad disease resistance. The smallest number of R lines was found against PM which poses a high risk owing to the rapid and easy spread of the causal pathogen. Likewise, this study provides relevant information about the needs of future breeding programs for this group of common beans and the potential genes and genomic regions to be considered for targeted improvement. A GWAS of the SBP showed the involvement of well-known chromosomal regions in the R responses against the four pathogens and identified new, or less characterized genomic regions that should be validated in future works. This highlighted the importance of characterizing different genetic resources, such as landraces and/or wild materials, to exploit their diversity in breeding. Breeding resistance to ANT in snap beans is mainly conducted using the Co-2 and Co-3 clusters. For future breeding programs, the use of other ANT resistance genes or clusters should be considered.

## Data availability statement

The datasets presented in this study can be found in online repositories. The names of the repository/repositories and accession number(s) can be found below: (https://www.ncbi.nlm.nih.gov/, PRJNA1076096).

## Author contributions

AC: Writing – original draft, Methodology, Formal analysis, Data curation, Conceptualization. VG: Writing – review & editing, Data curation. EB: Writing – review & editing, Data curation. AN: Writing – review & editing, Data curation. RP: Writing – review & editing, Supervision, Project administration, Funding acquisition. JF: Writing – review & editing, Supervision, Funding acquisition, Data curation.
