# Supplementary material for: Screening for resistance to four fungal diseases and associated genomic regions in a snap bean diversity panel
Source: Front Plant Sci. 2024 Jun 11;15:1386877. doi: 10.3389/fpls.2024.1386877 (PMC11196787; doi:10.3389/fpls.2024.1386877)
Supplement: Supplementary file 3 [file Presentation_1.pdf]

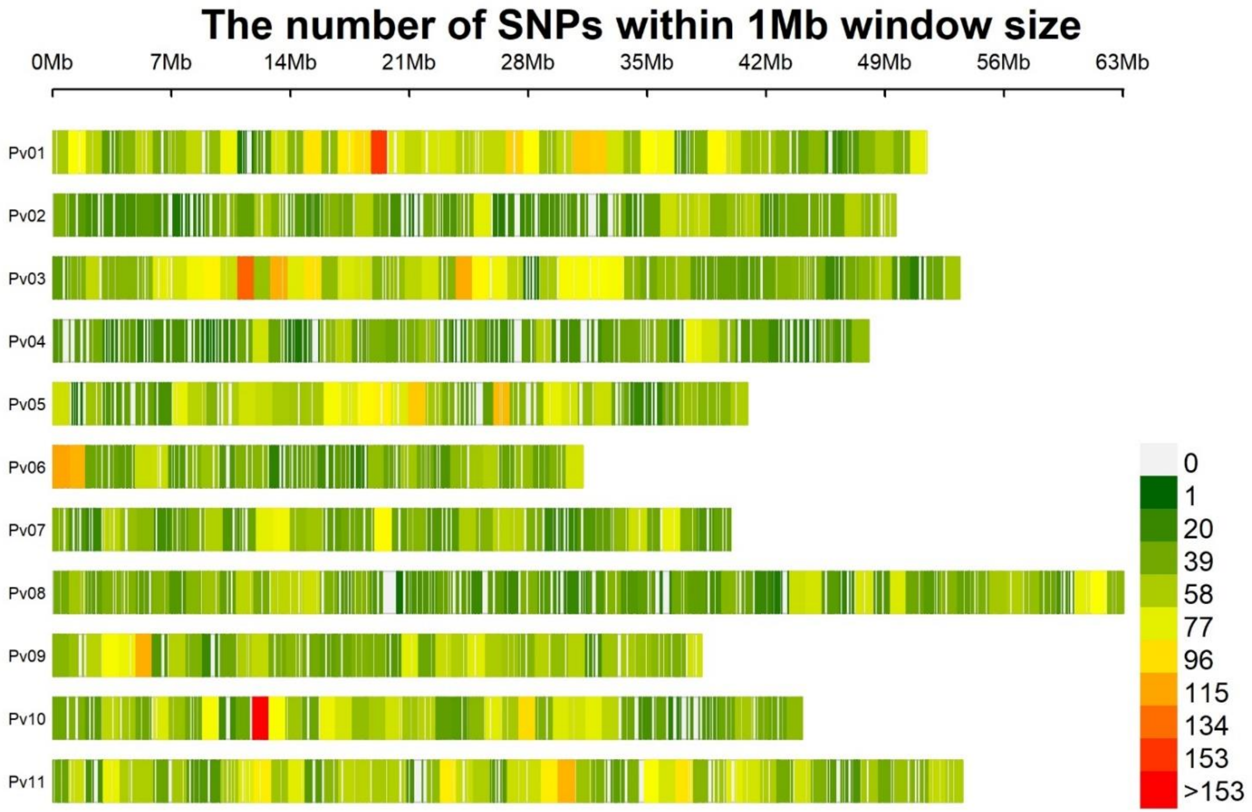

Supplementary Figure S1. Distribution along the eleven bean chromosomes of the 16,242 SNPs considered after filtering for the Genome-wide association study conducted in the Snap Bean Panel
